# A challenging case of a multiple large stones in enterocystoplasty: a case report

**DOI:** 10.11604/pamj.2024.48.116.43987

**Published:** 2024-07-18

**Authors:** Youssef Kadouri, Jihad Lakssir, Adam Elaboudi, Hachem El Sayegh, Yassine Nouini

**Affiliations:** 1 Department of Urology, Regional Hospital Center of Guelmim, Guelmim, Morocco,; 2 Department of Urology A, University Hospital Center, Rabat, Morocco

**Keywords:** Bladder tumor, enterocystoplasty, complications, stone, case report

## Abstract

In recent years, the utilization of enterocystoplasty for bladder function enhancement has increased, albeit accompanied by risks such as stone formation, necessitating vigilant follow-up. We report a case of a 60-year-old female with a neobladder who presented with back pain, constipation, and pelvic heaviness, revealing multiple large stones in imaging. Stone analysis showed calcium oxalate and magnesium. Enterocystolithotomy was performed to manage the condition. Enterocystoplasty, a standard treatment for bladder cancer, can lead to stone formation due to factors like urinary stasis and infections. Giant stones are rare but require prompt treatment, often involving neocystolithotomy. Controlling risk factors and selecting appropriate treatment based on stone size and surgical expertise are key to improving patient outcomes.

## Introduction

In recent years, the utilization of enterocystoplasty and related procedures for enhancing continence or replacing bladder function has surged. The risk of various complications, with stone formation being one of them, makes this a procedure requiring rigorous follow-up. Stone formation following enterocystoplasty may be present with different clinical presentations, however, it is more often asymptomatic. However, in exceptional cases, a stone can grow to a significant size, necessitating a prompt management that often leads to a surgical intervention [1]. We report a case of a 60-year-old female revealing multiple large stones in an enterocystoplasty secondary to an atypical clinical presentation.

## Patient and observation

**Patient information:** a 60-year-old female, with a history of passive smoking, underwent a radical cystectomy with enterocystoplasty for invasive bladder cancer 10 years ago.

**Clinical findings:** the postoperative follow-up was unremarkable for the first 7 years. However, the patient was lost to follow-up thereafter. She was readmitted to the urological emergency department, 3 years after her last check-up, due to bilateral lower back pain, constipation, and a sensation of pelvic heaviness. On clinical examination, the patient was in good general condition, with slight tenderness in the lumbar fossae. A hard mass was palpable in the hypogastric region, which was immobile. This mass compressed the rectum upon pelvic examination.

**Timeline:** ten years ago: radical cystectomy with enterocystoplasty for invasive bladder cancer. First 7 years post-surgery: unremarkable follow-up. Last 3 years: lost to follow-up. Present admission: bilateral lower back pain, constipation, pelvic heaviness.

**Figure 1:**
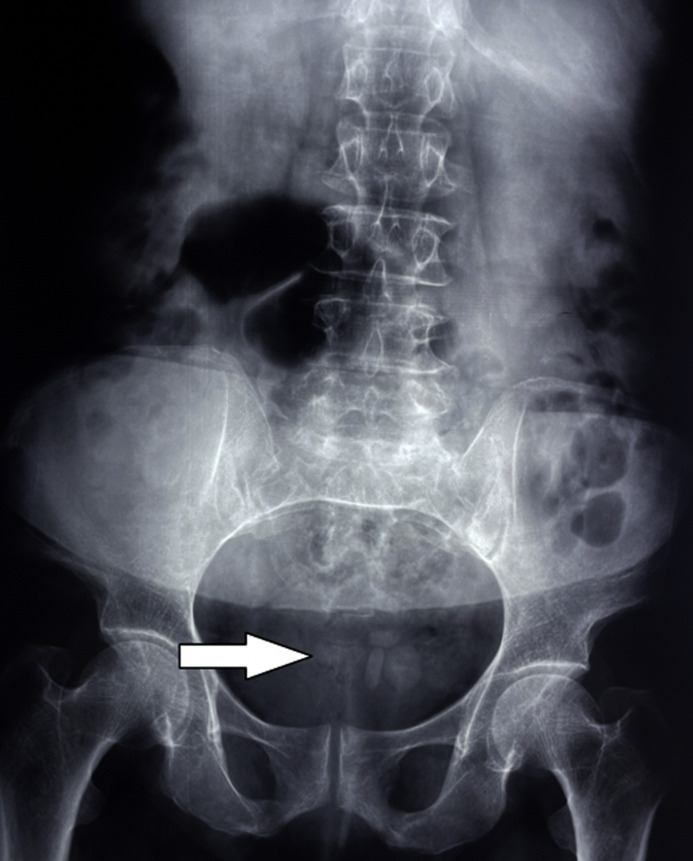
multiple opacities in the vesical area

**Figure 2:**
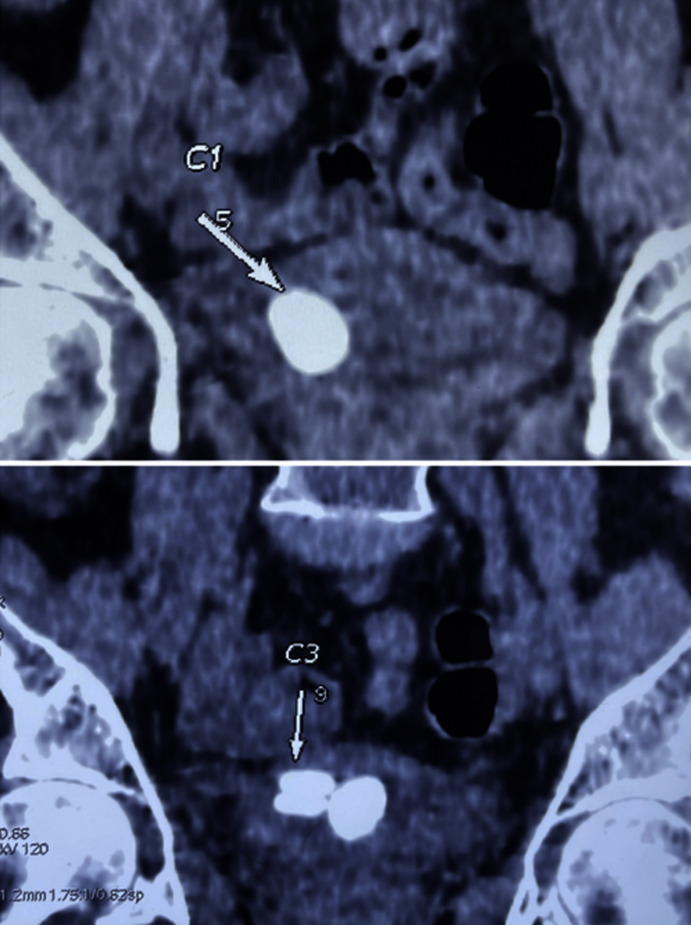
frontal section of a CT-Scan showing multiple neobladder stone

**Diagnostic assessment:** an abdominal X-ray revealed multiple unexpected opacities in the pelvic region (Figure 1). A CT scan followed, revealing large multiple stones within the neobladder, the largest measuring 3 cm (Figure 2). A laboratory assessment did not show any signs of renal insufficiency, infection, or inflammation. The urine culture revealed a urinary tract infection caused by *Escherichia coli*.

**Figure 3:**
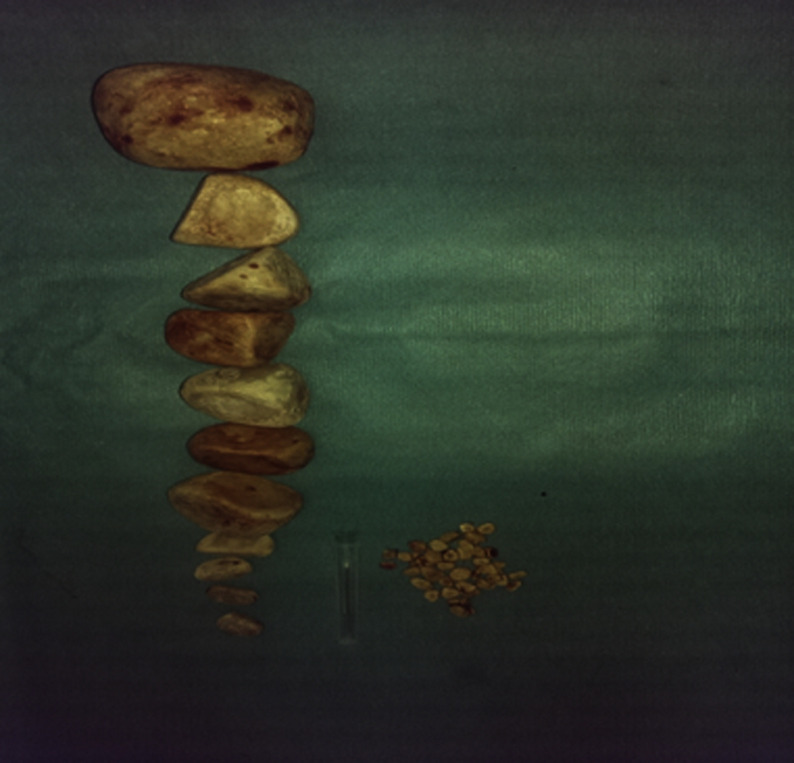
large stones after their extraction from the neobladder

**Therapeutic intervention:** after appropriate antibiotic therapy, the patient underwent an enterocystolithotomy, during which multiple large stones were extracted (Figure 3). Spectrophotometric analysis showed that the stones were primarily composed of calcium oxalate and magnesium. The metabolic assessment for lithiasis showed no particularities.

**Follow-up and outcomes:** the patient showed a good clinical course, and follow-up was unremarkable.

By reporting this observational case and performing a review of the literature according to the CARE recommendations (using the PubMed database and guidelines from urology, general surgery and oncology societies), we present the epidemiology, clinical presentation of enterocystoplasty stones, diagnostic means, and therapeutic options. We used the following keyword associations in French and English: “Enterocystoplasty”, “Enterocystoplasty stones”, “Enterocystoplasty complications" and “Bladder cancer”.

**Informed consent:** written informed consent was obtained from all the patients for the publication of this study and accompanying images.

## Discussion

Since the 1950s, radical cystectomy with enterocystoplasty or the Bricker procedure has been considered the gold standard in the treatment of invasive bladder cancer [2]. Long-term complications are well-documented and include metabolic disturbances such as hyperchloremic metabolic acidosis, bone density loss, recurrent infections, renal deterioration, and urolithiasis [3]. The meta-analysis conducted by Mathoera *et al*. in 2000 demonstrated a prevalence ranging from 18.2% to 52.5%, with an average 38 months after surgery of [4]. However, the occurrence of giant bladder stones remains an exceptional complication; only 5 cases have been reported in the literature, with the largest weighing 940 grams [5].

Several factors are implicated in the formation of stones on enterocystoplasty, such as urinary stasis, recurrent urinary tract infections and mucus secretion [6]. Another risk factor for the occurrence of lithiasis is the presence of foreign bodies within the bladder, they contribute to stone formation in 13% to 43% of cases [7]. Many patients with bladder stones exhibit no symptoms, even in the presence of very large stones. Some may report finding small stone fragments in their urine during catheterization or pouch flushing. Recurrent infection is often the most characteristic presentation of bladder stones [8]. Regarding the positive diagnosis, clinical examination is often limited and nonspecific. Urinary tract radiography and abdominal ultrasound establish the diagnosis in 95% of cases. Intravenous urography or CT urography enables a comprehensive exploration of the entire urinary tract and specific analysis of the stone: localization, number, size, density, and impact on the upper urinary tract.

The treatment of stones on enterocystoplasty depends on the size of the stones and the type of urinary diversion used. However, neocystolithotomy remains the treatment of choice for large stones [9], as was the case for our patient. Other therapeutic modalities can be considered such as endoscopic laser lithotomy and percutaneous lithotomy. For extracorporeal shock wave lithotripsy, its effectiveness remains limited in stones on enterocystoplasty [10].

## Conclusion

The occurrence of giant stones on enterocystoplasty remains an exceptional complication. Controlling risk factors is a beneficial approach that can improve the quality of life for these patients. Treatment primarily depends on the size of the stone and the experience of the surgeon.
